# Development and Validation of a New Multidimensional Measure of Inspiration: Associations with Risk for Bipolar Disorder

**DOI:** 10.1371/journal.pone.0091669

**Published:** 2014-03-26

**Authors:** Steven Jones, Alyson Dodd, June Gruber

**Affiliations:** 1 Spectrum Centre for Mental Health Research, Faculty of Health & Medicine, Lancaster University, Lancaster, United Kingdom; 2 Department of Psychology, Yale University, New Haven, Connecticut, United States of America; Catholic University of Sacred Heart of Rome, Italy

## Abstract

**Background:**

Individuals at risk for, and diagnosed with, bipolar disorder (BD) appear to have heightened levels of creativity. Although inspiration is creativity, the ways in which individuals appraise and respond emotionally to inspiration in BD remain unexplored.

**Method:**

The present study reports on a new measure of inspiration (External and Internal Sources of Inspiration Scale - EISI). The reliability and validity of EISI were explored along with associations between EISI and BD risk.

**Results:**

Among a cross-national student sample (*N* = 708) 5 inspiration factors were derived from EISI (self, other, achievement, prosocial and external inspiration). Reliability, concurrent validity and convergent/divergent validity were good. Total EISI and all subscales were associated with increased positive rumination, and total EISI and the achievement EISI subscale were associated with impulsivity. Total EISI, self and prosocial EISI subscales were independently associated with BD risk and current mania symptoms.

**Conclusion:**

This new measure of inspiration is multidimensional, reliable and valid. Findings suggest that self and prosocial focused inspiration are particularly associated with risk for BD after controlling for current manic symptoms. Future studies in clinical populations may illuminate the relationships between inspiration and creativity in BD.

## Introduction

Inspiration is increasingly being investigated as a psychological construct encompassing emotional and motivational factors [1]. However there is as yet little information on how individuals appraise and respond to inspiration experiences. Understanding more about inspiration is important because it is a key aspect of creativity [2], [3] which itself is highly associated with mental health problems, in particular bipolar disorder [4], [5]. This paper describes the development of a new measure which fills this gap in the literature. It also explores the extent to which appraisals of, and emotional responses to, inspiration are associated with risk for bipolar disorder.

### Creativity and Risk for Bipolar Disorder (BD)

Bipolar disorder (BD) is associated with heightened levels of achievement and creativity [4], [5] and with positive cognitive styles, including positive self-appraisal, positive rumination, impulsivity, achievement motivation and elevated goal setting (e.g. [6], [7], [4], [8], [9], [10]). However, research directly exploring the cognitive and emotional substrates of creativity and its relationships with BD is currently lacking. This is despite a robust literature highlighting the unique association between creativity and BD. This was originally identified through biographical accounts of artists, musicians, poets and writers whose records indicate experiences of mania and depression diagnostic of BD [11], [12], [13], [14], [15]. More recently, evidence has been found for heightened levels of creativity among non-eminent individuals with BD [16], [5], [17]. Furthermore, the relationships observed between creativity and achievement in BD are apparent even in those with milder presentations of BD, as well as those at risk for developing BD in the future (e.g., [16], [17]). An example of the evidence for creativity in BD comes from a study by Richards et al. [18] which demonstrated that higher levels of lifetime creative achievements (including entrepreneurial, artistic or scientific activity) were observed in individuals with BD spectrum disorders and first-degree BD relatives compared with healthy controls. High level vocational accomplishments identified by Richards and colleagues were evenly distributed across occupational domains.

Individuals with BD report creative experiences as a highly valued positive aspect of their condition [19].This is clinically relevant because perceived threats to creativity through treatment can be associated with adherence issues [20]. In particular, a failure to understand this aspect of BD experiences by clinicians could lead to lack of engagement with therapies if the client fears that clinical improvement might be associated with losses of creativity. In contrast, harnessing the positive aspects of creativity might inform new treatment approaches, help engagement and adherence with therapy, and improve therapy outcomes [21], [20], [22], [23]. Although creativity is an important issue in BD spectrum conditions, the core processes that underpin creativity in BD are unclear and a direct exploration of its underlying emotional and cognitive architecture is warranted.

### Linking Creativity with Emotion and Positive Cognitive Style in BD

Definitions of creativity suggest the importance of emotional processes. One emotional/motivational process linked to creativity is inspiration, which is associated with generating ideas that can be translated into creative outputs [2], [3]. Attempts to measure inspiration have been largely dominated by work by Thrash and colleagues [1] who posit a tripartite definition of inspiration. Accordingly, inspiration is conceptualized as an psychological construct which includes i) transcending ordinary possibilities; ii) is evoked by an object, agent or underlying process; and iii) is associated with approach motivation to realise the idea or possibility so evoked. From this model of inspiration emerged a one-dimensional measure of inspiration frequency and intensity, shown to correlate with measures of creativity [24], [25], [1]. Increased levels of inspiration on this measure are higher in US patent holders – emblems of creative output – compared to a control group of university alumni [24].

Importantly, inspiration experiences have been highly associated with positive affective states [24]. Although links between inspiration and BD risk have not been directly assessed, a growing body of work suggests heightened positive emotion is a core feature of BD and BD risk [26]. Specifically, BD is associated with heightened positive emotion reactivity, e.g., [27], [28]. For example, individuals with BD report greater positive affect (PA) in response to daily life events [29] and emotional photos and films [30], [31], [32]. Moreover, BD individuals have difficulties down-regulating positive emotions [33], and exhibit a tendency to up-regulate or intensify existing positive moods [34], [35]. Increases in positive mood promote increased approach motivation towards pursuing goal-driven behaviour [6], [36], [9]. In sum, increased positive mood states are associated with both inspiration and BD, both highly related to, and potentially fuelling, creative output.

A second important facet of creativity involves positive cognitive styles [22]. In this vein, BD is associated with increased positive self-appraisals following minor elevations in mood [37], [8], [38]. For example, using a factor analysis of multiple positive cognitive measures, Johnson & Jones [39] found that overly positive appraisals of hypomanic symptoms were strongly associated with BD risk. Thus, individuals who make such appraisals have an elevated tendency to explain increases in energy, creativity, or motivation as reflecting something fundamental about their own abilities and powers. This contrasts with individuals with low BD risk who are more prone to explaining changes in these variables as being associated with external environmental factors. Self-relevant beliefs about needing to be ‘on-the-go’ to avert failure are also associated with increased activation in BD [40]. Under these conditions people with BD are also less likely to take advice and more likely to work towards increasing ambitious goals [7], [27], [41]. This pattern can lead to both high levels of achievement, as noted above, and also to clinical episodes of mania in BD [42]. Consistent with this potential pattern of escalation from initial appraisal into symptomatic states, characteristic response styles have also been reported in bipolar disorder when in the context of positive affect. These response styles include rumination on the self and emotional experiences, tendencies towards impulsive action, and generation of new ideas [43], [44], [45]. Such response styles are also associated with the positive cognitive styles characteristic of BD [37], [43], [39]. The importance of a pattern of internally driven positive appraisals, achievement focus and characteristic response styles has also been recognised in the clinical therapy literature on BD. For instance, Lam et al.'s [46] evidence-based cognitive therapy manual highlights the importance of training individuals in the associations between external events and mood experiences as a pre-requisite for successful engagement in relapse prevention work. It would therefore be expected that if inspiration experiences are relevant to BD, a measure of inspiration would exhibit relationships to the characteristic ruminative and impulsive response styles to positive affect also observed in BD.

### The Present Investigation

Research to date indicates the importance of creativity in BD, and potential links between such experiences and positive emotion and positive cognitive styles. However, the emotional and cognitive underpinnings of creativity in BD have yet to be fully explored. One promising avenue is through the lens of inspiration which is closely tied with both positive emotion and cognition states. In light of these findings, the current study sought to evaluate the ways in which inspiration is experienced and appraised in individuals at risk for BD, and to explore the ways in which such experiences are associated with related mood symptoms and response styles to positive mood. Given previous findings regarding the importance of internal versus external appraisals in BD (e.g., [8] etc.), we also wanted to investigate the extent to which beliefs people hold about the source or trigger of inspiration experiences influenced relationships with mood and response style variables. As there is no currently established measure designed to do this, we report on the EISI (External and Internal Sources of Inspiration) measure. The EISI was designed to explore beliefs about inspiration, namely about the source of inspiration (i.e., as experienced when generated by self, others, or the wider environment) and to capture the role of behavioural and emotional sequelae of inspiration.

The present study therefore sought to develop a measure of different facets of inspiration to help guide future research on the cognitive and emotional underpinnings of BD and in the field more generally. We achieve this through two broad aims. Our first aim was to describe the new EISI measure in some detail and report on an initial evaluation of its psychometric characteristics including factor structure, internal reliability, and associations with related measures of rumination and impulsivity. In line with this aim, we tested two main hypotheses. Hypothesis 1 focused on the development and validation of EISI and predicted that: (1a) EISI scale will be internally reliable and associated with an established unidimensional measure of inspiration; (1b) Inspiration from internal and external sources will be psychometrically distinguishable; and (1c) Inspiration experiences (measured by EISI) , in particular higher levels of self-generated inspiration experiences, will be associated with ruminative and impulsive response styles (measured by Responses to Positive Affect and Positive Urgency Measures respectively; detailed in Method section below) for positive affect.

The second aim was to test theory-driven hypotheses regarding associations between this measure and BD risk. We predicted that: (2a) Inspiration experiences in general will be associated with elevated BD risk; and (2b) more specifically, self-focused inspiration experiences will be more strongly positively associated with BD risk than externally focused inspiration experiences, given the importance of self-relevant appraisals and risk for BD as outlined above [8].

## Methods

### Ethics Statement

Written informed consent was taken at both sites. At Lancaster University, all participants gave online consent via Survey Monkey before proceeding to the study measures. At Yale University, all participants provided written informed consent to participate in this study, including electronic confirmation or written signature on consent forms. All research including consenting procedures was conducted in accordance with approvals received from the Lancaster University Research Ethics Committee and by the Yale University Institutional Review Board.

### Participants

Undergraduate students (*N = *835) were recruited to complete online questionnaires from both Yale University in the U.S. (*n = *510) and Lancaster University in the U.K. (*n = *324).

### Self-Report Questionnaires (see also Table 1)

**Table 1:** Description of Questionnaire Measures.

*Construct*	*Measure*	*Subscales*	*Scale Length*	*Cronbach's Alpha (Current Study)*
*BD-Risk*	*HPS*	*N/A*	48	0.86
*Mania symptoms*	*ASRM*	*N/A*	5	0.74
*Inspiration*	*IS*	*Intensity*	4	0.87
		*Frequency*	4	0.86
	*EISI*	*Self-focus*	9	0.87
		*Other-focus*	4	0.86
		*Achievement-focus*	5	0.80
		*Prosocial-focus*	3	0.84
		*External-focus*	4	0.60
*Rumination*	*RPA*	*Self-focused Positive Rumination*	4	0.76
		*Emotion-Focused Positive Rumination*	5	0.72
		*Dampening*	8	0.82
*Impulsivity*	*PUM*	*N/A*	14	0.96

*Note:* Hypomanic Personality Scale (HPS); Altman Self Rating Mania Index (ASRM); Inspiration Scale (IS); External and Internal Scale of Inspiration (EISI); Responses to Positive Affect Scale (RPA); Positive Urgency Measure (PUM).

#### Clinical Measures

BD risk: The Hypomanic Personality Scale (HPS) [47] is a widely-used and well-validated 48-item self-report measure of BD risk, capturing episodic shifts in emotion, behavior, and energy. The HPS has excellent predictive validity for the onset of manic/hypomanic episodes, e.g. [47], [48]. Previous research further indicates that scores on the HPS correlate with clinical diagnoses of bipolar disorder e.g., [47] and current or life time mania symptoms [49].

Current mania symptoms: Current symptoms of mania were assessed using the 5-item self-report Altman Self-Rating Mania Scale (ASRM) [50], measuring heightened cheerfulness, inflated self-confidence, reduced need for sleep, talkativeness, and excessive activity. These items load onto a single component in factor analyses that is highly correlated with both clinical interview and self-report measures of mania [51]. Scores ≥14 indicate clinically significant levels of current manic symptoms.

#### Inspiration Measures

Inspiration Scale (IS) [24]. IS items are: ‘I experience inspiration’, ‘something I encounter or experience inspires me’, ‘I am inspired to do something’, and ‘I feel inspired’. Each of the items are rated in terms of Intensity (‘How deeply or strongly in general’) on a 1 (not at all) to 7 (very strongly) scale, and Frequency (‘How often does this happen?’) on a 1 (never) to 7 (very often) scale. Both the Intensity and Frequency subscales had good internal consistency in the original paper (α = .94 and .91, respectively).

**Figure 1:**
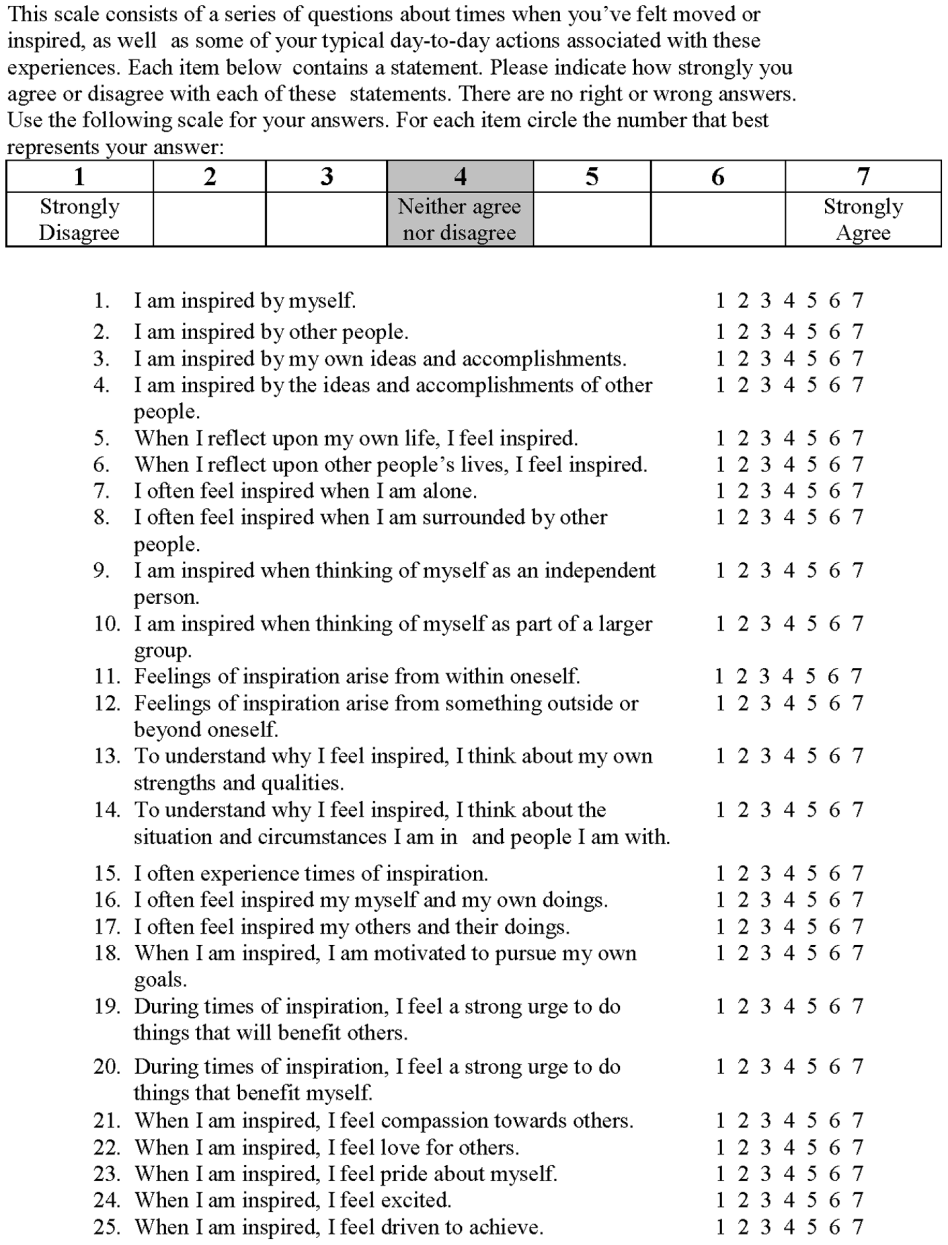
External and Internal Scale of Inspiration (EISI).

External and Internal Scale of Inspiration (EISI). The EISI was developed for the present investigation as a measure of external and internal inspiration relevant to experiences of those with BD. The EISI consists of 25 total items rated on a 7-point Likert scale from 1 (*strongly disagree*) to 7 (*strongly agree*). For a full list of scale items see Table 2. Initial items for the EISI were brainstormed by authors (SJ and JG), both of whom are experienced clinical psychologists and researchers in the psychology of BD. As such, items were based on clinical experience as well as driven by models of BD, particularly achievement-focus and the internality/externality of positive cognitive styles known to be associated with bipolar disorder [7], [27], [42], [8], [28]. Based on these sources the EISI items reflected sources of inspiration (internal/external), contexts of inspiration (social/solitary), rationale for inspiration (internal/external) drive associated with inspiration (social/achievement), and feelings associated with inspiration (love, pride, excitement, compassion). Items were phrased to match the intensity and frequency question format of the original IS scale [24]. The final version of the EISI is presented in Figure 1 and is also available from the corresponding author.

**Table 2:** Component loading table for External and Internal Inspiration Scale.

*Item*	*Factor*					*h^2^*	*r_i(t-i)_*	*M*	*sd*	*Corrected α*
	1	2	3	4	5					
***16.***	**.831**	−.015	.114	.000	.141	.72	.59	4.26	1.43	.88
***1.***	**.792**	.024	.159	.037	.150	.68	.60	4.50	1.51	.88
***3.***	**.771**	.032	.162	.061	.182	.66	.62	4.80	1.39	.88
***5.***	**.686**	.155	.138	−.022	.228	.57	.58	4.28	1.52	.88
***15.***	**.640**	.206	.098	.134	.034	.48	.54	4.58	1.46	.88
***11.***	**.621**	−.024	.178	.106	−.131	.45	.41	5.00	1.39	.88
***7.***	**.585**	.200	.067	.085	−.180	.43	.40	4.39	1.59	.88
***9.***	**.557**	.118	.246	.087	.036	.39	.50	5.03	1.44	.88
***13.***	**.541**	−.189	.194	.050	.368	.50	.46	4.37	1.59	.88
***4.***	.057	**.827**	.173	.085	.132	.74	.45	5.73	1.19	.88
***2.***	.068	**.801**	.113	.115	.163	.70	.43	5.75	1.20	.88
***6.***	.104	**.794**	.096	.114	.174	.69	.45	5.24	1.33	.88
***17.***	.141	**.747**	.039	.104	.269	.66	.47	5.16	1.19	.88
***25.***	.157	.187	**.746**	.188	.004	.65	.50	6.05	1.02	.88
***18.***	.238	.224	**.718**	.177	−.049	.66	.54	6.00	1.08	.88
***20.***	.185	−.016	**.715**	−.186	.121	.59	.38	5.26	1.38	.88
***24.***	.181	.161	**.662**	.252	.046	.56	.51	6.05	1.02	.88
***23.***	.339	−.038	**.641**	.089	.197	.57	.52	5.35	1.40	.88
***21.***	.127	.083	.114	**.871**	.143	.81	.44	5.37	1.38	.88
***22.***	.132	.101	.154	**.813**	.163	.74	.45	5.31	1.33	.88
***19.***	.054	.186	.086	**.791**	.112	.68	.38	5.37	1.37	.88
***14.***	.037	.141	.073	.086	**.658**	.47	.32	4.87	1.32	.88
***10.***	.186	.127	−.069	.113	**.609**	.44	.34	4.37	1.50	.88
***12.***	−.071	.179	.114	.072	**.597**	.41	.25	4.66	1.44	.89
***8.***	.180	.210	.073	.119	**.576**	.43	.41	4.69	1.37	.88

*Note: Factor* 1 = Self-focus; Factor 2 = Others-focus; Factor 3 = Achievement-focus; Factor 4 = Emotion = focus; Factor 5 = External-focus h^2^ = Communality; r_i(t-i) = _Corrected item-total correlation; Corrected α = Cronbach's α if item delete.

#### Measures to Obtain Divergent and Convergent Validity for the EISI

Two additional measures were chosen to obtain divergent and convergent validity for the EISI that measured ruminative and impulsive responses to positive affect, respectively, which have been robustly documented in adults at risk for, and diagnosed with, BD [43], [44], [45].

Responses to Positive Affect Scale (RPA) [44]. The RPA measures rumination and dampening in response to positive affect. The scale comprises 3 subscales: Dampening (e.g., Think “I don't deserve this”’, α = 0.72); Self-focused Positive Rumination (e.g., ‘Think “I am achieving everything”’, α = 0.73); and Emotion-Focused Positive Rumination (e.g., ‘Think about how strong you feel’, α = 0.76).

Positive Urgency Measure (PUM) [52]. The PUM measures tendency to respond impulsively to positive affect. Items (e.g., ‘When I am very happy, I tend to do things that may cause problems in my life’) are rated on a Likert scale from 1 (*agree strongly*) to 4 (*disagree strongly*), and reversed before summing total scores. Internal consistency was α = 0.94 in the original development paper [52].

### Procedure

At Lancaster University in the U.K., undergraduate students were invited to take part in an online study on ‘Personality and the Student Experience’ via posters, flyers, and a mass email sent to the student body. At Yale University in the U.S., sessions were also conducted with undergraduate students using an anonymous online survey in exchange for partial course credit. Across both sites, participants first provided informed consent. Next, they completed the above measures and some additional measures not relevant to the current study, using Survey Monkey (at Site 1, Lancaster University) or Qualtrics (at Site 2, Yale University). At Lancaster University, additional catch items were added (e.g., ‘Please select ‘Strongly agree’ for this question’). Sessions lasted up to 60 minutes total.

### Data Analysis Strategy

Participants were removed from the U.K. data where they had responded to ‘catch items’ incorrectly. These were inserted at random intervals throughout the online questionnaire battery, and participants were instructed to give a specific response e.g., ‘Please respond ‘4’ here’. Data across the two sites were then merged. Merged data was screened and in this process participants were excluded if they: i) had duplicate student ID numbers; ii) had given impossible answers to items, for example responded >7 where the maximum response was 7 (US data only); or iii) had not completed the External and Internal Scale of Inspiration (EISI). Descriptive statistics (mean, standard deviation, minimum and maximum) were generated using SPSS.

To explore the latent themes underlying the EISI for Hypothesis 1a, we conducted a Principal Components Analysis (PCA) with Varimax rotation. Parallel analysis was used to ascertain the number of components to retain using SPSS syntax available online [53], [54], from which 1,000 random datasets were produced, with 708 cases and 25 variables.

To test Hypothesis 1b, we examined the psychometric properties of the EISI. Reliability was assessed using Cronbach's alpha. Concurrent validity was assessed by comparing the EISI with a more established measure of inspiration, the IS [24]. Partial correlations were conducted to test whether associations between the EISI and the IS were significant when controlling for current symptoms of mania.

To test Hypothesis 1c, we examined divergent and convergent validity for the EISI. Specifically, Pearson's correlations were conducted between the EISI and positive response styles associated with achievement motivation and BD. Partial correlations were conducted to test whether associations between response styles, and inspiration were still significant even when controlling for current symptoms of mania.

To test Hypothesis 2a, associations between the EISI and the HPS and ASRM were explored by conducting bivariate correlations. Partial correlations were conducted to test whether associations between response styles, inspiration, and bipolar risk were still significant even when controlling for current symptoms of mania.

To test Hypothesis 2b, a series of hierarchical multiple regressions were conducted to explore i) whether HPS and ASRM predicted the different facets of inspiration (EISI subscales) and if current symptoms (ASRM) moderated the relationship between inspiration and vulnerability to BD (HPS) and ii) specific associations between EISI subscales and vulnerability to BD (HPS). Durbin-Watson statistics were all >1 and <3, confirming that the assumption of independent error was tenable. Based on the VIF and Tolerance statistics, there were no concerns about multicollinearity. Plots did not indicate any concerns about homoscedasticity, and standardised residuals were normal. For each regression model, Cook's distance, Mahalanobis distance, and the Centred Leverage Values were examined for potential outliers. Two cases were removed from these analyses as they exceeded the recommendations on these values, and they were found to have an undue influence on the regression models (Field, 2005).

To address (i) above, regressions were run with each EISI subscale as the outcome variable, we standardised the scores for the predictor variables and computed the interaction as a product of standardised *z*-scores on the ASRM and HPS. For each regression model, standardised scores for the ASRM and HPS were entered in Step 1. In Step 2, the interaction term ‘ASRM * HPS’ was entered to assess its unique contribution to the variance.

Next, to address (ii) above, those EISI subscales significantly associated with HPS scores were entered together into a further hierarchical multiple regression analysis to explore the unique variance accounted for by each subscale, after controlling for ASRM scores. In the regression model, ASRM was entered in Step 1. In Step 2, the EISI subscales were entered to assess their unique contribution to the variance.

The *n* for each comparison varies as SPSS removes cases automatically from analyses pertaining to specific measures where the participant does not have a score for that measure.

## Results

### Preliminary Analyses

**Figure 2:**
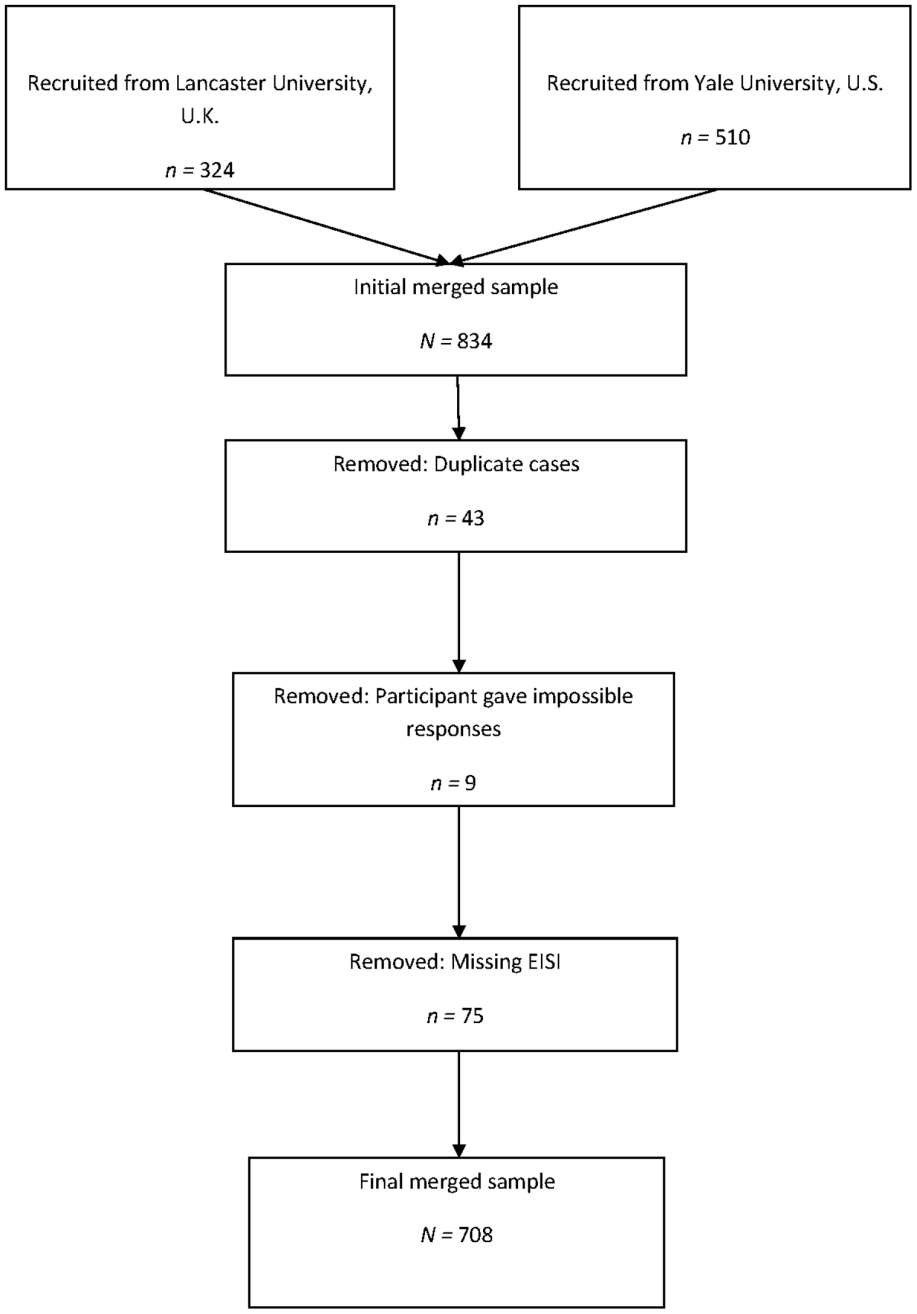
Participant flow chart.

Participant flow is illustrated in Figure 2. The final sample (*N = *708) had a mean age of 20.59 years (*SD = *19.35), and 455 (64.2%) were female. Descriptive statistics for all questionnaires are shown in Table 3. For all measures, skewness and kurtosis were acceptable (i.e., values not substantially greater than zero, and within the limits of skewness <2 and kurtosis <7 [55], [56]). Thus the assumption of normality for multivariate analyses was tenable.

**Table 3:** Descriptive Statistics for all variables.

*Variable*	*N*	*M*	*SD*	*Min*	*Max*
***HPS***	706	17.26	8.12	0	44
***ASRM***	690	5.36	3.60	0	17
***RPA Self-focus***	695	9.44	2.67	4	16
***RPA Emotion-focus***	695	13.69	2.93	5	20
***RPA Dampening***	695	14.79	4.56	8	31
***PUM***	655	2.79	.78	1	4
***IS Intensity***	702	4.42	1.08	1.25	7
***IS Frequency***	702	4.70	1.07	1.25	7
***EISI overall***	708	5.06	.70	2.24	6.88
***Self-focus***	708	4.58	1.03	1	7
***Others-focus***	708	5.47	1.03	1	7
***Achievement-focus***	708	5.77	.88	1.4	7
***Prosocial-focus***	708	5.35	1.16	1	7

Hypomanic Personality Scale (HPS); Altman Self Rating Mania Index (ASRM); Responses to Positive Affect Scale (RPA); Positive Urgency Measure (PUM); Inspiration Scale (IS); External and Internal Scale of Inspiration (EISI).

### Main Analyses: Hypothesis Testing

#### Aim 1: Establish Psychometric Properties of EISI

Hypothesis 1a. Inspiration from internal and external sources will be psychometrically distinguishable. In the parallel analysis, five components had actual eigenvalues greater than the mean and 95^th^ percentile random data eigenvalues (see Table S1). In addition, in the PCA output, 5 components had eigenvalues greater than one, and the scree plot also levelled out at 5 factors. For all items on the EISI, skewness and kurtosis were acceptable, with values not substantially greater than zero, and within the limits of skewness <2 and kurtosis <7 [55], [56]. The Kaiser-Meyer-Olkin statistic was .874, and verified sampling adequacy. Bartlett's test was *X* ^2^ (300) = 7515.66, *p*<0.001, indicating the items correlated sufficiently for PCA. The 5-component solution accounted for 58.77% of variance. Table 2 shows the component loadings for each item after rotation. Component 1 (Self-focus) accounted for 18.14% of the variance (eigenvalue = 4.54), and included items reflecting being inspired from within yourself, and your own achievements, strengths and ideas. Component 2 (Others-focus) accounted for 11.8% of the variance (eigenvalue = 2.95), and included items related to being inspired by others' lives and achievements. Component 3 (Achievement-focus) accounted for 11.15% of the variance (eigenvalue = 2.80), and included items relevant to feeling motivated, excited and goal-oriented in response to inspiration. Component 4 (Prosocial-focus) accounted for 9.41% of the variance (eigenvalue = 2.35), and included items pertaining to feeling compassionate, loving and altruistic towards others when feeling inspired. Component 5 (External-focus) accounted for 8.28% of the variance (eigenvalue = 2.07), and included items reflecting beliefs about feeling inspired due to social factors and the wider environment, and was labelled External-focus. In sum, findings were consistent with hypothesis 1a by demonstrating a clear distinction between self-focused inspiration and inspiration from other sources (other-focus and external-focus). An additional distinction revealed by PCA was between responses to feeling inspired, in particular between achievement orientated and prosocial reactions to inspiration.

Hypothesis 1b. The EISI scale will be internally reliable and associated with an established unidimensional measure of inspiration. Reliability was excellent for the overall scale (Cronbach's α = .89) and the subscales Self-focus (α = .87) , Others-focus (α = .86), Achievement-focus (α = .80), and Prosocial-focus (α = .84). Cronbach's α for External-focus was adequate (α = .60) and thus no items were recommended for deletion. Correlations between the EISI and the IS are given in Table 4. The EISI and all EISI subscales were positively and significantly correlated with IS Intensity and Frequency. Partial correlations, controlling for ASRM were conducted, and the pattern of results remained the same. In sum, these results are consistent with hypothesis 1b.

**Table 4:** Correlations between the EISI, EISI subscales with IS, RPA, PUM, HPS and ASRM.

Variable	EISI	Self	Others	Achievement	Prosocial	External
**Hypothesis 1b: correlations with IS**						
IS Intensity	.56**	.57**	.33**	.30**	.23**	.28**
IS Frequency	.50**	.47**	.26**	.33**	.24**	.24**
**Hypothesis 1c: Correlations with RPA and PUM**						
RPA Dampening	−.06	−.15**	.05	−.08	.12*	.02
RPA Self-focus	.40**	.41**	.09	.36**	.14**	.18**
RPA Emotion-focus	.36**	.28**	.16**	.28**	.30**	.20**
PUM	.12*	.10	.03	.15**	.03	.06
**Hypothesis 2a: Correlations with HPS and ASRM**						
HPS	.28**	.27**	.07	.20**	.23**	.12*
ASRM	.25**	.23**	.11*	.17**	.17**	.12*

*Note:* EISI = External and Internal Scale of Inspiration; IS = Inspiration Scale; HPS = Hypomanic Personality Scale; ASRM = Altman Self Rating Mania Index; RPA = Responses to Positive Affect Scale; PUM = Positive Urgency Measure; Hypothesis 1b: ** *p<*0.001 (Adjusted α-level after Bonferroni correction = 0.004); Hypothesis 1c: *p<0.002 **p<0.001 (Adjusted α-level after Bonferroni correction = 0.002); Hypothesis 2a: *p<0.003 **p<0.001 (Adjusted α-level after Bonferroni correction = 0.003).

Hypothesis 1c. Inspiration experiences, in particular, higher levels of self-generated inspiration experiences will be associated with ruminative and impulsive response styles for positive affect. Results are displayed in Table 4. The overall EISI and all EISI subscales had significant positive relationships with RPA Self-focused Positive Rumination, with the exception of Others-focused Inspiration. Emotion-focused Positive Rumination was associated with all subscales. Self-focused Inspiration was negatively associated with RPA Dampening, while Prosocial-focused Inspiration was positively associated with Dampening. The overall EISI and Achievement-focused Inspiration were positively associated with the PUM. The pattern of results remained the same when controlling for ASRM scores. In sum, consistent with Hypothesis 1c, ruminative responses (self and emotion focused) and impulsivity, were associated with overall EISI score. All subscales of EISI were also associated with rumination expect for the lack of a relationship between Other-focused inspiration and self-focused rumination. The relationships between EISI subscales and impulsivity were less strong, with only Achievement-focused inspiration significantly associated.

#### Aim 2: Associations between EISI and BD risk

Hypothesis 2a. Inspiration experiences in general will be associated with elevated BD risk. Results (see Table 4) revealed that the EISI and most subscales showed small significant correlations with the HPS and ASRM. The pattern of positive relationships between the EISI and EISI subscales (Self, Achievement, Prosocial, and External-focus) and the HPS remained significant when controlling for the ASRM in a partial correlation. This pattern of relationships is consistent with Hypothesis 2a.

Hypothesis 2b. Self focused inspiration experiences will be more strongly positively associated with BD risk than externally focused inspiration experiences. To test Hypothesis 2b, two sets of regression analyses were then computed to explore relationships between EISI and BD risk in more detail. Regression models met required assumptions (see Data Analysis). Results are given in Table 5 (*n = *689) and Figure S1. Specifically, ASRM and HPS were both independently associated with overall EISI and Self-focused Inspiration. However, only BD risk (HPS) was associated with Achievement and Prosocial Inspiration, independently of current symptoms. Other than the EISI overall, Self-focused Inspiration appears to have the most robust association with BD risk (current mania symptom and trait vulnerability). Thus it appears that the facets of inspiration most related to bipolar vulnerability are about self-focused high-achievement, and less about elevations in pro-social sources or the influence of others. In no case was the interaction between ASRM and HPS a significant associate of inspiration score.

**Table 5:** Hypothesis 2b: Associations between HPS, ASRM, and EISI subscales.

Variable	EISI	Self	Others	Achievement	Prosocial	External
	B	β	β	B	B	β
**Step 1**						
ASRM	.19**	.16**	.11	.13**	.11	.09
HPS	.22**	.22**	.03	.16**	.20**	.09
**Step 2**						
ASRM	.33**	.30**	.25	.17	.12	.18
HPS	.32**	.31**	.13	.20**	.20**	.15
ASRM x HPS	−.21	−.21	−.20	−.07	.15	−.13

Note: EISI = External and Internal Scale of Inspiration; HPS = Hypomanic Personality Scale; ASRM = Altman Self Rating Mania Index. EISI, R^2^ = .11** at step 1, ΔR^2^ = .004 at step 2.

Self, R^2^ = .10** at step 1, ΔR^2^ = .004 at step 2.

Others, *R* ^2^ = .02** at step 1, *ΔR* ^2^ = .004 at step 2.

Achievement, *R* ^2^ = .06** at step 1, *ΔR* ^2^ = .001 at step 2.

Prosocial, *R* ^2^ = .06** at step 1, *ΔR* ^2^ = .000 at step 2.

External, *R* ^2^ = .02** at step 1, *ΔR* ^2^ = .002 at step 2.

*p<0.003.

**p<0.001 (Adjusted α for Bonferroni correction = .003).

Second, having established the associations between HPS and each aspect of the EISI, we also wished to establish the relative explanatory power of the respective EISI subscales. EISI Self-focused Inspiration and Prosocial Inspiration were significantly independently associated with HPS in a positive direction, even when controlling for ASRM (see Table 6 and Figure S2). In sum, results are consistent with Hypothesis 2b that the self as source of inspiration was significantly associated with BD risk in contrast with other-focused/external inspiration subscales. Additionally, the only other unique associate of BD risk was prosocial inspiration that was not specifically predicted. These two subscales also differed in that only Self-focused inspiration was also associated with mania symptoms.

**Table 6:** Hypothesis 2b: Associations between EISI subscales and bipolar risk, when controlling for current mania.

Variable	HPS
**Step 1**	
ASRM	.33**
**Step 2**	
ASRM	.27**
Self	.16**
Others	−.07
Achievement	.05
Prosocial	.15**
External	.01

Note: *R* ^2^ = .11** at step 1, *ΔR* ^2^ = .06** at step 2. HPS = Hypomanic Personality Scale; ASRM = Altman Self-Rating Mania Scale.

**p<0.001 (Adjusted α for Bonferroni correction = .003).

## Discussion

Creativity has been repeatedly linked with BD and there is increasing evidence for inspiration as playing an important role in the expression of creativity. However, to date there have been no studies directly exploring the cognitive and emotional underpinnings of creativity in relationship to BD. This paper reports on the development of a new measure (EISI) designed to explore the different facets of inspiration experienced in BD spectrum conditions based on prior research and clinical experience. This area of research is important given that creativity is highly valued by individuals with BD and is relevant to treatment adherence, e.g., [20], [22], [19].

### Aim 1: Psychometric Properties of the EISI

Our first aim was to establish the psychometric properties of EISI as a valid and reliable measure of distinct facets of inspiration experience. There was broad support consistent with our hypotheses in relation to this first aim with respect to a stable factor structure, internal reliability and divergent and convergent validity. The findings also suggest that the relationships between inspiration and positive response styles are more complex than would be apparent from a unitary measure of inspiration. In particular other-focus inspiration does not seem to be linked to response styles observed in BD. Overall these findings suggest the EISI scores are significantly associated with behavioural tendencies that could have clinical significance, as both rumination and impulsiveness have been associated with worse clinical outcomes in BD [57], [58], [10]. Additionally, the different patterns of association for different inspiration subscales, supports the proposal that inspiration itself is multifaceted.

### Aim 2: Associations between EISI and BD risk

Having established the initial psychometric properties of the EISI, our second aim was to evaluate the relationship between EISI and its subscales and risk for BD. There was broad support consistent with our hypotheses in relation to this second aim as well.

There was a consistent pattern of associations between EISI and its subscales and both bipolar risk (except for other focused inspiration) and current manic symptoms. The former remained significant after controlling for current mania symptoms. These associations were particularly strong for EISI total and the self-inspiration sub-scale, and non-significant for other-focused/external inspiration. A second regression confirmed the importance of self-focused and prosocial inspiration as unique associates of BD risk, even when controlling for mood. Although a consistent pattern of significant associations was identified the effect sizes were modest. This indicates that although inspiration and bipolar risk are linked it is important to explore other variables to more fully understand bipolar risk as discussed below.

### Implications for the Study of BD Risk

This initial paper was primarily focused on the development of the EISI as a BD relevant measure of inspiration. As yet we have not explored the relationships between patterns of inspiration and creativity in general, although this is an obvious direction for future research. Studies of analogue populations indicate that inspiration and creativity measures are associated [24]. In addition, inspiration seems to share a number of associations in common with measures of positive cognitive style previously explored in bipolar disorder. Thus, positive self-appraisal, heightened reward responsiveness and approach motivation have all been associated with increased manic symptoms, and with patterns of impulsive and ruminative responding (as observed for inspiration in the current paper). Inspiration has also been shown to be associated with changes in positive affect and elevated approach motivation, which are also observed in bipolar disorder [59], [60], [24], [25], suggesting that exploration of the relative contributions of inspiration and positive cognitive style might have merit in understanding more about the psychology of bipolar risk. One possible reason for these overlapping associations might be that inspiration is not a distinguishable concept from aspects of positive cognitive style in general. However, this seems unlikely given the recent series of studies which have indicated specific links between creative ideas and inspiration. More specifically, an investigation of creativity in poetry and fiction writing indicated that inspiration served as a specific mediator between the creativity of the original idea and the creativity of the final output [1]. Furthermore, although univariate associations were found between inspiration and PA, approach motivation, effort and openness, SEM modelling including these variables supported a specific transmission pathway between creative ideas, inspiration and creative output only. Additionally, Milyavskaya and colleagues [61] reported a significant relationship between trait inspiration and achievement of key personal goals over a three month period in a student sample. Individual difference variables accounted for 65% of variance in inspiration, with extraversion and conscientiousness having particularly strong links with inspiration. It is of note that extraversion has been shown to be elevated in bipolar compared to unipolar disorder [62] and predictive of future bipolar disorder in healthy young men [63].

### Limitations and Future Directions

There are some significant limitations to the current research that it is important to acknowledge: First, this was a cross-sectional study so we do not yet have any data on prospective associations between inspiration and mood or response style. Second, this initial study was conducted in student samples to explore associations with risk for bipolar disorder. We used a measure of behavioural high-risk [43] rather than a semi-structured diagnostic interview or assessing familial risk of BD. Although previous research has supported the applicability of such risk research to clinical bipolar samples e.g. [8] it remains untested whether the specific patterns of inspiration observed here will also be observed in those with a clinical diagnosis of bipolar disorder. In addition, although the present design used a well-validated measure of BD risk, future studies are warranted that example bipolar risk through the use of a long-term prospective study designs or larger epidemiological designs. Specifically, it is unknown whether any of our sample had experienced an episode of (hypo)mania. However, as noted by Johnson and colleagues [22], some of the clearest associations between bipolar and creativity have been in individuals at risk or with milder forms of the disorder, so the current sample is appropriate from that perspective. Also, by studying a large cross-national sample, it is hoped that the associations observed will be both replicable and applicable to future studies. Thirdly, we relied solely on self- report measures and did not directly assess creative outputs, both of which could be addressed in future research in this area.

In understanding more about the role of inspiration in bipolar disorder, it would be of interest to assess whether the mediating role observed for inspiration in analogue research [1] is apparent in individuals with bipolar spectrum conditions, and which aspects of inspiration serve this role most prominently. Refining our understanding of the role of inspiration in bipolar disorder is also of more than academic interest. As indicated by the current study, inspiration is associated with ruminative and impulsive response styles that have potentially important clinical implications. Furthermore, inspiration appears to be importantly related to the wider concept of creativity, which Johnson and colleagues [22] have noted may influence the ways in which people engage (or not) with current evidence based therapies.

## Conclusions

Our new measure of inspiration indicates that it is multidimensional with self and prosocial focused inspiration particularly associated with risk for hypomania after controlling for current manic symptoms in a cross-national student sample. The current findings suggest that inspiration is worthy of further investigation in bipolar disorder, including prospective studies in clinical samples to clarify the significance of the cross-sectional relationships observed in this report. Further research exploring the relationships between measures of creativity and inspiration in bipolar disorder are also indicated.

## Supporting Information

Figure S1 **Scatterplots for associations between bipolar risk, mania, and their interaction with EISI subscales.**
(TIFF)

Figure S2 **Scatterplots for associations between EISI subscales and bipolar risk, when controlling for current mania.**
(TIF)

Table S1 **Random data eigenvalues and 95^th^ percentile eigenvalues for parallel analysis of EISI.**
(DOCX)
