# Supplementary figures and images for: Development and Validation of a New Multidimensional Measure of Inspiration: Associations with Risk for Bipolar Disorder
Source: PLoS One. 2014 Mar 26;9(3):e91669. doi: 10.1371/journal.pone.0091669 (PMC3966762; doi:10.1371/journal.pone.0091669)

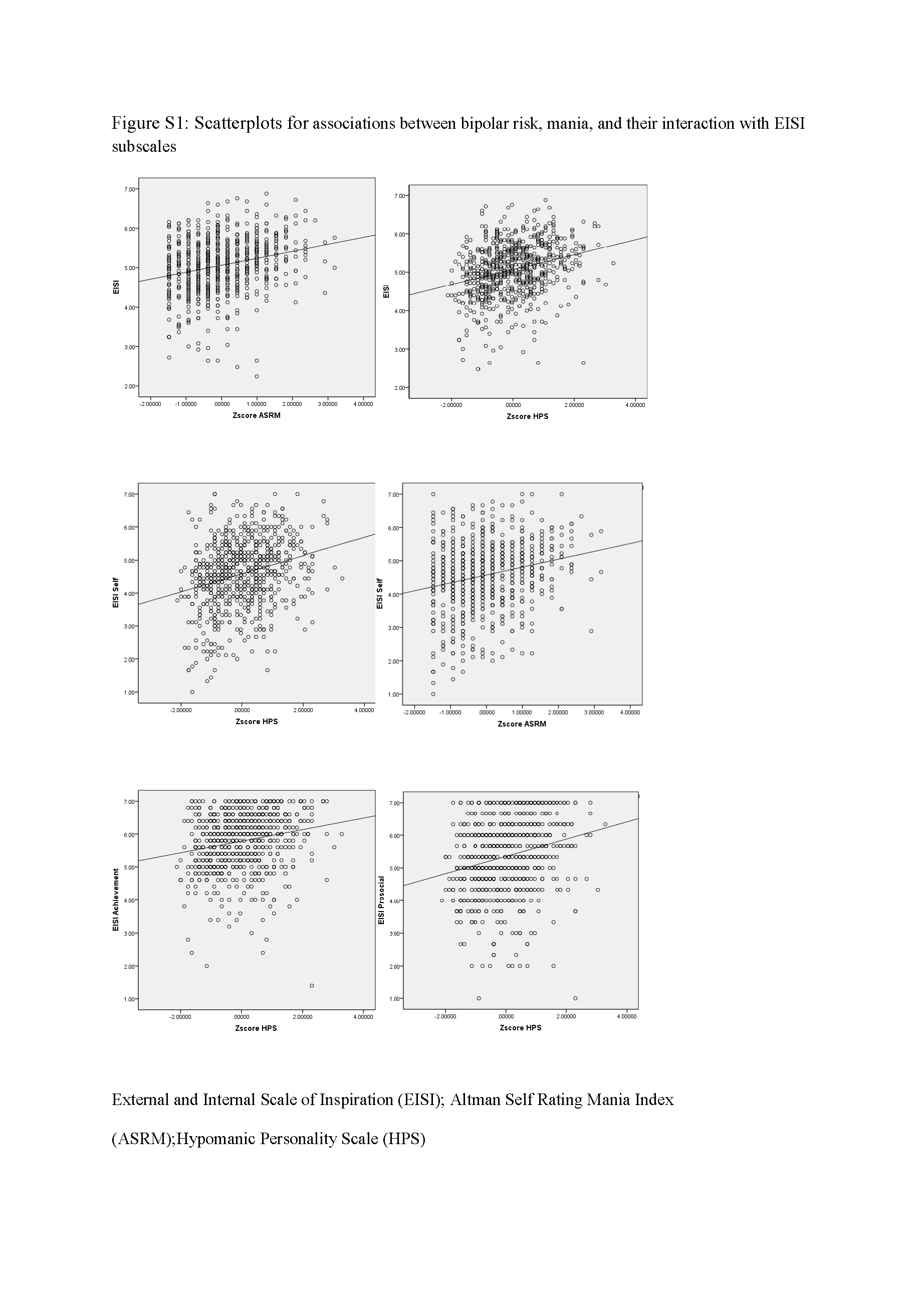

Supplement: Figure S1 — Scatterplots for associations between bipolar risk, mania, and their interaction with EISI subscales. (TIFF) [file pone.0091669.s001.tif]

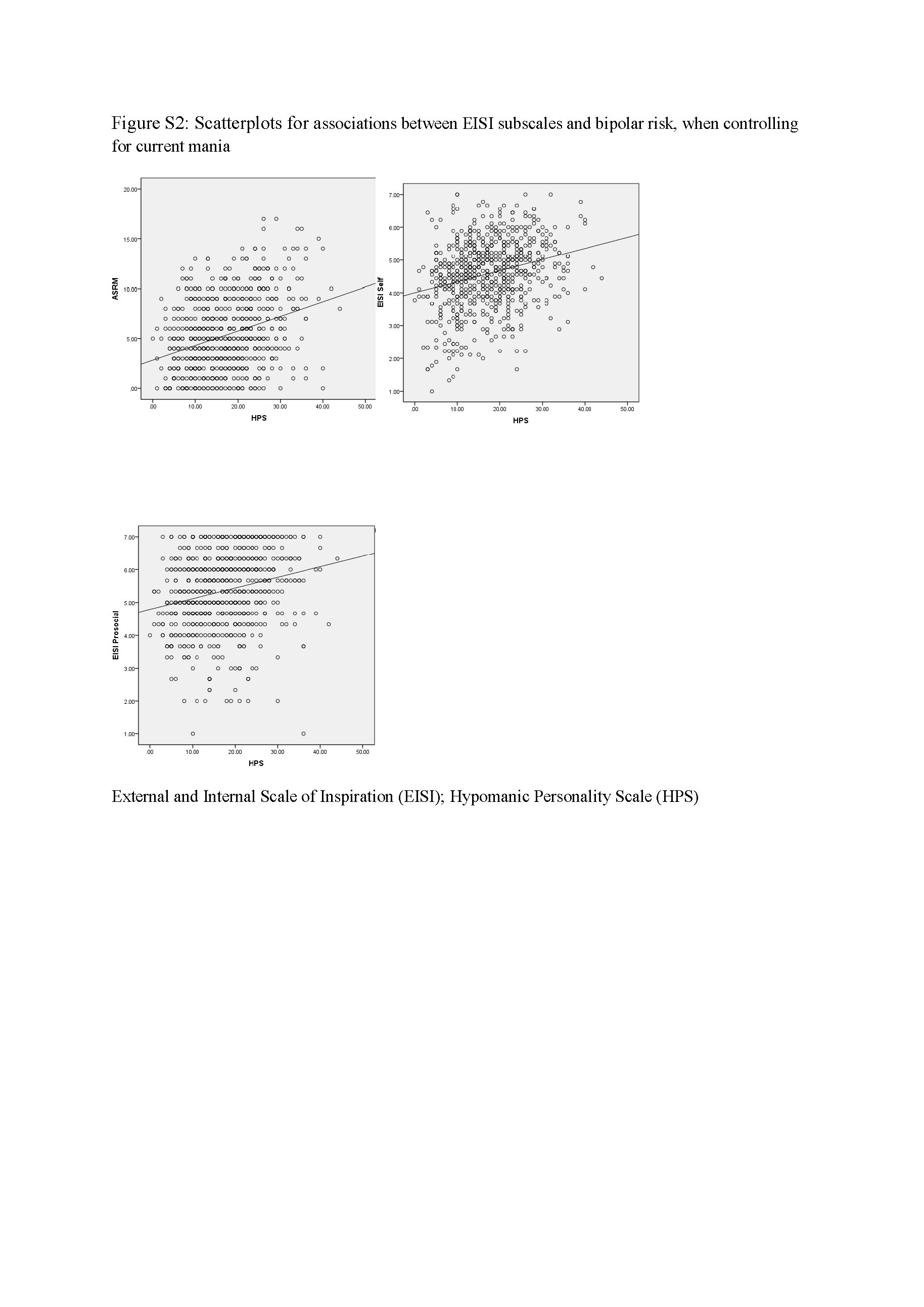

Supplement: Figure S2 — Scatterplots for associations between EISI subscales and bipolar risk, when controlling for current mania. (TIF) [file pone.0091669.s002.tif]
