# Supplementary material for: Development and Validation of a New Multidimensional Measure of Inspiration: Associations with Risk for Bipolar Disorder
Source: PLoS One. 2014 Mar 26;9(3):e91669. doi: 10.1371/journal.pone.0091669 (PMC3966762; doi:10.1371/journal.pone.0091669)
Supplement: Table S1 — Random data eigenvalues and 95th percentile eigenvalues for parallel analysis of EISI. (DOCX) [file pone.0091669.s003.docx]

**Table S1.** Random data eigenvalues and 95^th^ percentile eigenvalues for parallel analysis of EISI

| Component | Initial eigenvalues | Mean | 95^th^ percentile |
| --- | --- | --- | --- |
| 1 | 6.95 | 1.36 | 1.41 |
| 2 | 2.96 | 1.30 | 1.34 |
| 3 | 1.81 | 1.26 | 1.30 |
| 4 | 1.66 | 1.23 | 1.25 |
| 5 | 1.32 | 1.19 | 1.22 |
| 6 | .88 | 1.16 | 1.19 |
| 7 | .86 | 1.14 | 1.16 |
| 8 | .80 | 1.11 | 1.34 |
| 9 | .77 | 1.08 | 1.11 |
| 10 | .74 | 1.06 | 1.08 |
| 11 | .70 | 1.04 | 1.06 |
| 12 | .64 | 1.01 | 1.03 |
| 13 | .62 | .99 | 1.10 |
| 14 | .53 | .97 | .99 |
| 15 | .53 | .94 | .96 |
| 16 | .49 | .92 | .94 |
| 17 | .41 | .90 | .92 |
| 18 | .38 | .88 | .90 |
| 19 | .36 | .85 | .88 |
| 20 | .33 | .83 | .85 |
| 21 | .30 | .81 | .83 |
| 22 | .27 | .78 | .81 |
| 23 | .26 | .76 | .78 |
| 24 | .23 | .73 | .75 |
| 25 | .22 | .69 | .72 |

Figure S1: Scatterplots for associations between bipolar risk, mania, and their interaction with EISI subscales


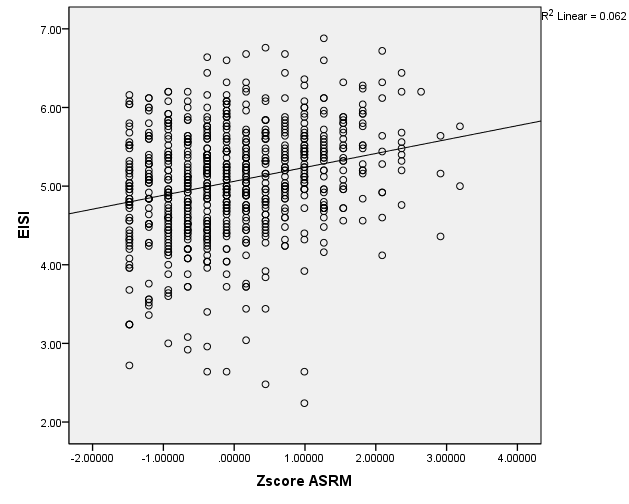

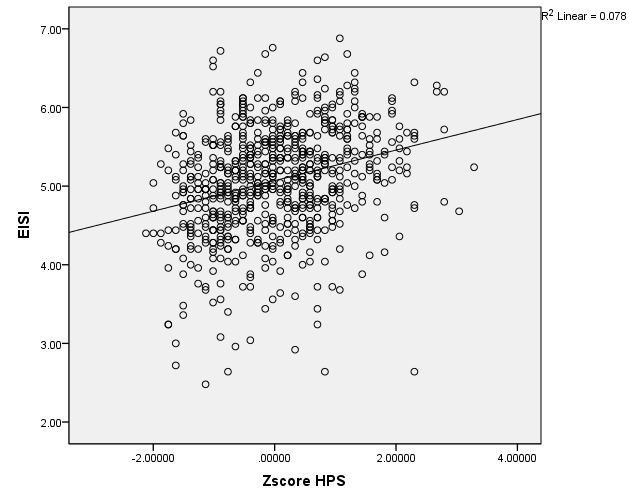


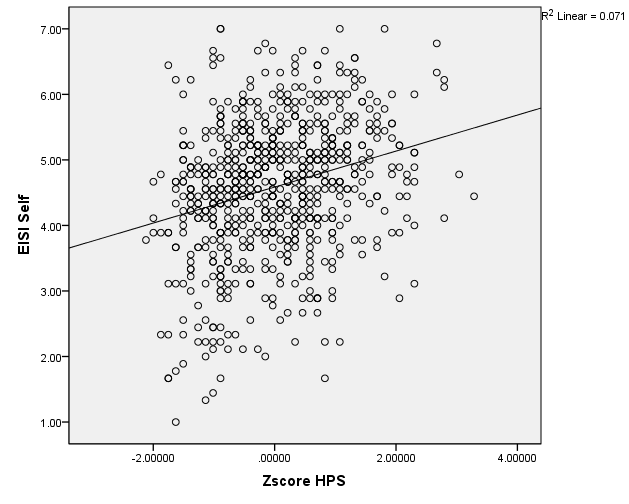

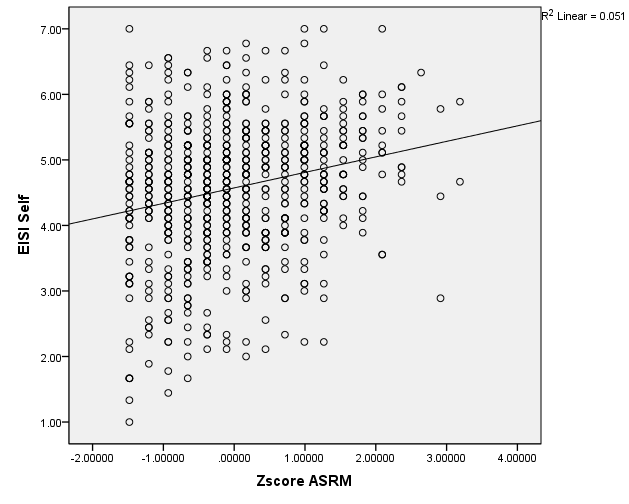


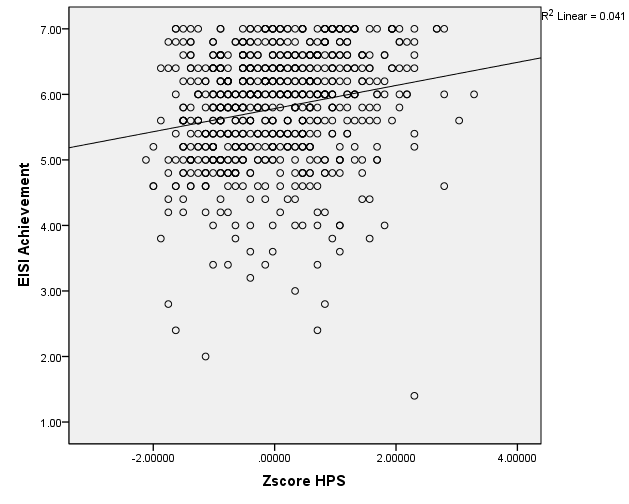

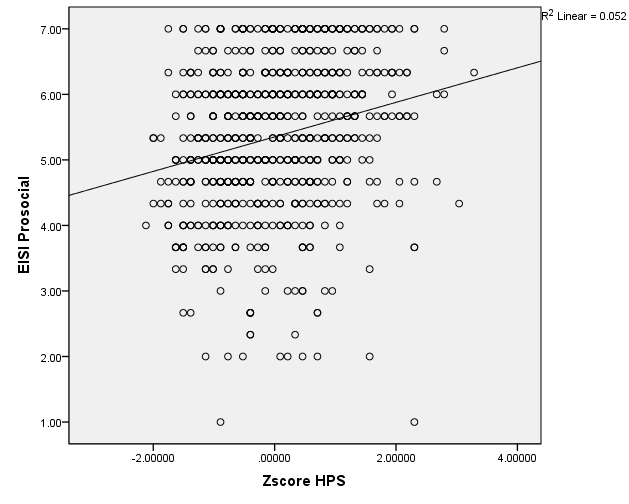


External and Internal Scale of Inspiration (EISI); Altman Self Rating Mania Index (ASRM);Hypomanic Personality Scale (HPS)

Figure S2: Scatterplots for associations between EISI subscales and bipolar risk, when controlling for current mania


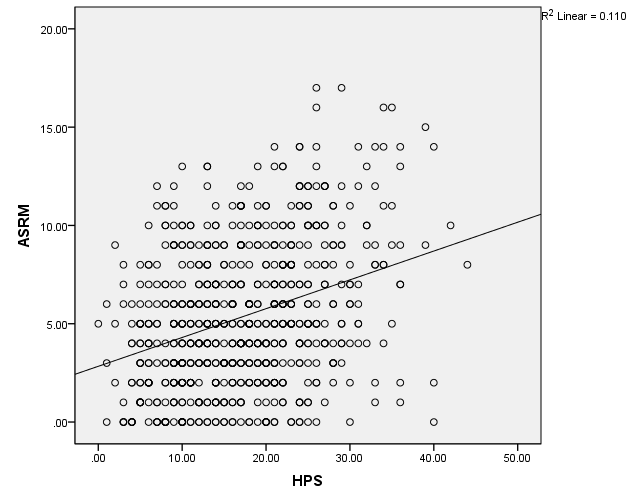

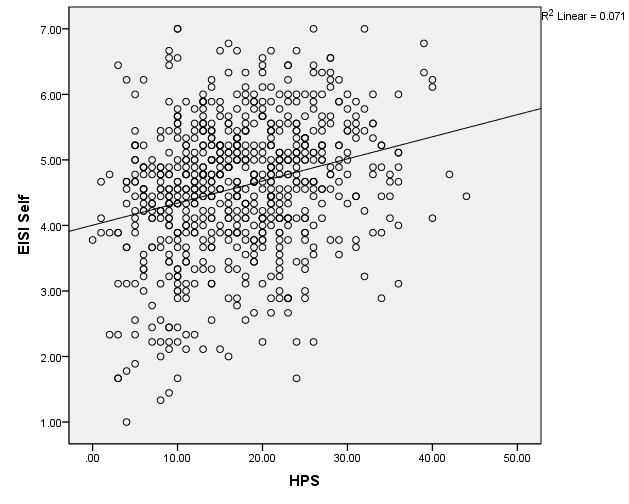


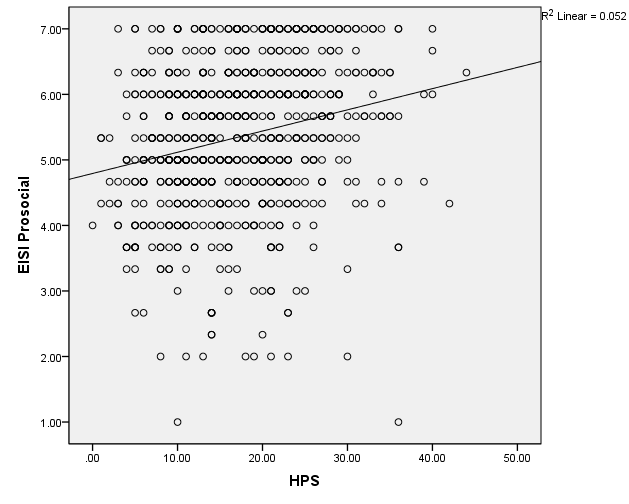


External and Internal Scale of Inspiration (EISI); Hypomanic Personality Scale (HPS)
